# Supplementary material for: Proliferative arrest induces neuronal differentiation and innate immune responses in normal and Creutzfeldt-Jakob Disease agent (CJ) infected rat septal neurons
Source: PLoS One. 2025 May 28;20(5):e0323825. doi: 10.1371/journal.pone.0323825 (PMC12118874; doi:10.1371/journal.pone.0323825)
Supplement: S1 Table — All genes are downregulated in at least 2 of the 3 samples (see text) with comparable fold changes (Log2Ratio columns). (DOCX) [file pone.0323825.s001.docx]

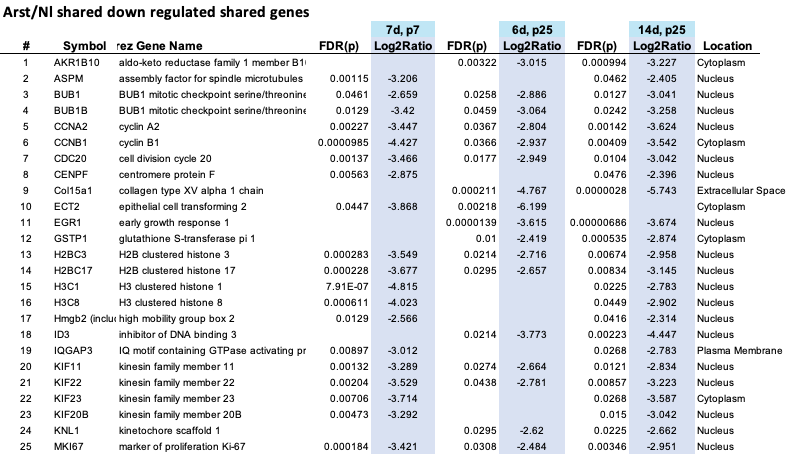


**S1 Table1:** **Top 25 genes down regulated in each of 3 independent samples from different passage of Nl cells,** i.e., Arst/Nl for 7 days from passage 7, at 6 days from p 25, and at 14 days from p25. All genes are downregulated in at least 2 of the 3 samples (see text) with comparable fold changes (Log_2_Ratio columns).
