# Supplementary material for: Proliferative arrest induces neuronal differentiation and innate immune responses in normal and Creutzfeldt-Jakob Disease agent (CJ) infected rat septal neurons
Source: PLoS One. 2025 May 28;20(5):e0323825. doi: 10.1371/journal.pone.0323825 (PMC12118874; doi:10.1371/journal.pone.0323825)
Supplement: S2 Fig — Interaction network built using STRING knowledgebase (v. 12.0). Network was imported into Cytoscape (v. 3.10.2) and clustering analysis was performed with MCODE (v. 2.0.3) with degree cutoff 2, node density cutoff 0.1, K-core 2, and maximum depth 100. Resulting clusters were functionally annotated on STRING database. (DOCX) [file pone.0323825.s002.docx]

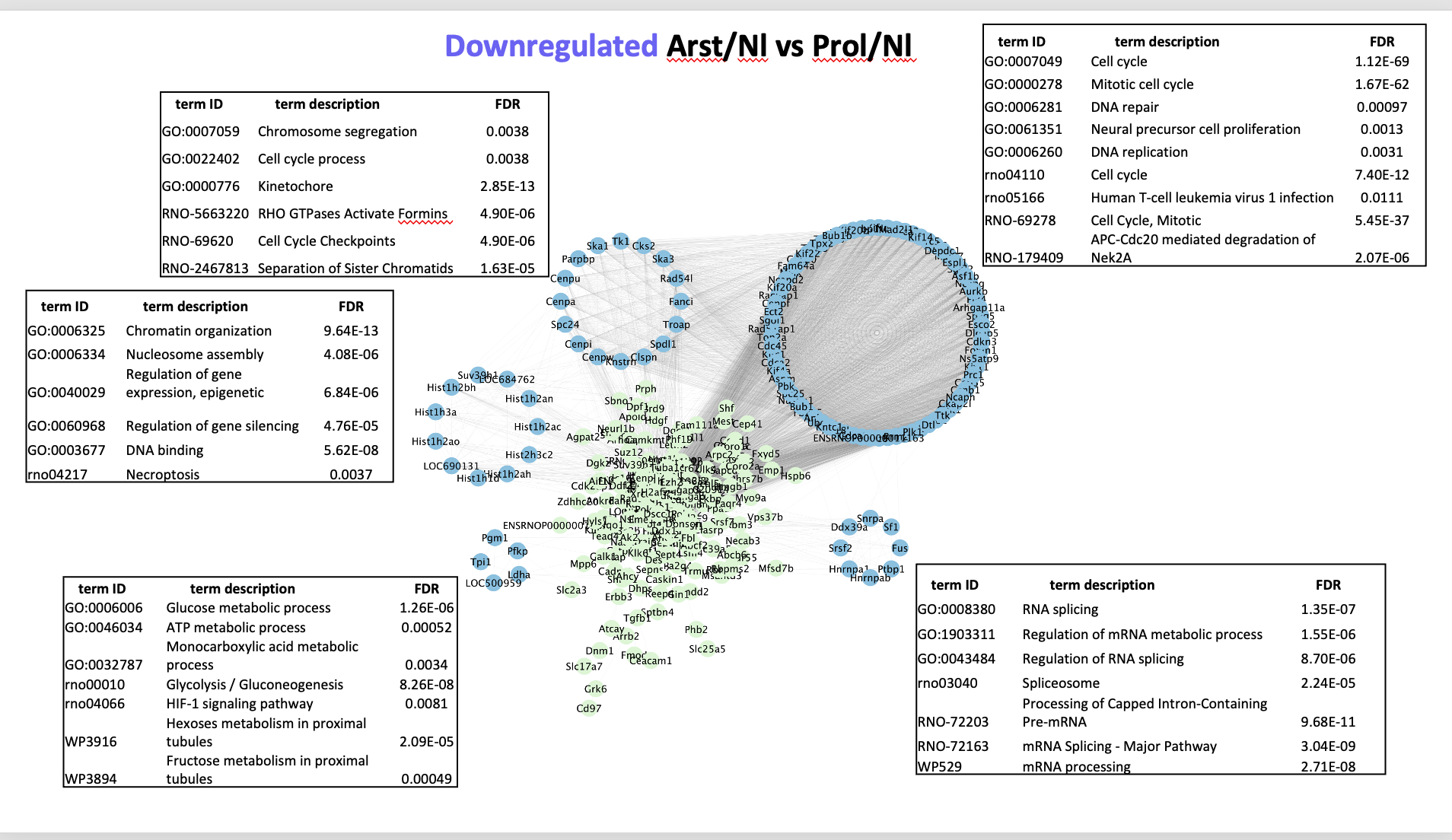


**S2 Fig.: Arts/Nl vs Prol/Nl: Interaction network analysis of differentially downregulated genes.** Interaction network built using STRING knowledgebase (v. 12.0). Network was imported into Cytoscape (v. 3.10.2) and clustering analysis was performed with MCODE (v. 2.0.3) with degree cutoff 2, node density cutoff 0.1, K-core 2, and maximum depth 100. Resulting clusters were functionally annotated on STRING database.
