# Supplementary material for: Proliferative arrest induces neuronal differentiation and innate immune responses in normal and Creutzfeldt-Jakob Disease agent (CJ) infected rat septal neurons
Source: PLoS One. 2025 May 28;20(5):e0323825. doi: 10.1371/journal.pone.0323825 (PMC12118874; doi:10.1371/journal.pone.0323825)
Supplement: S3 Fig — Upstream regulators network for IFNA2, IFNB1, and IFNG (A), and results from upstream regulators prediction and activation status. Intensity of red color in molecules indicates expression levels as shown in the legend. B) Table with prediction confidence and activation z-score value for each molecule. (DOCX) [file pone.0323825.s003.docx]

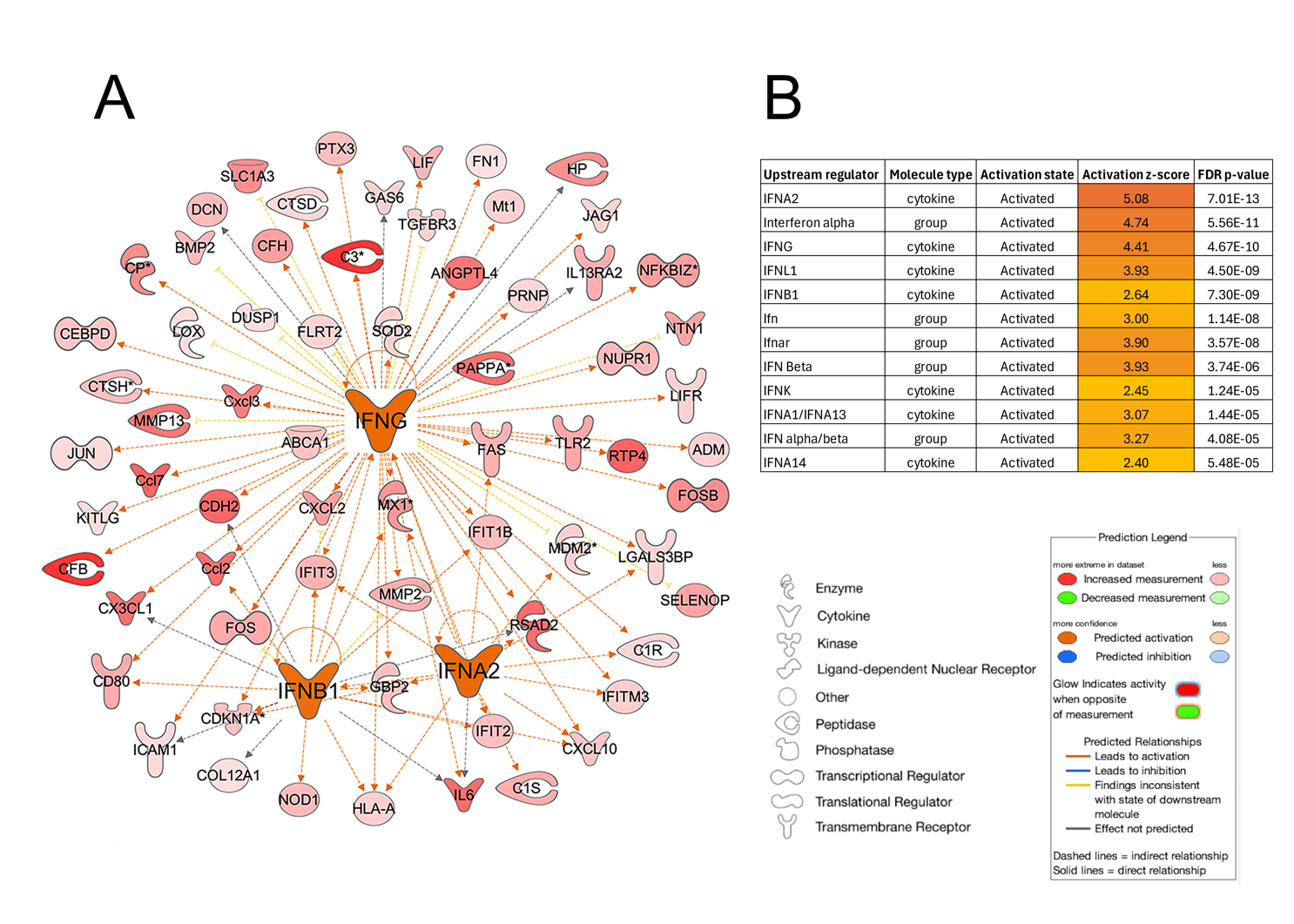


**S3 Fig.: Ingenuity Pathway Analysis for IFN in Nl neurons**. Upstream regulators network for IFNA2, IFNB1, and IFNG (A), and results from upstream regulators prediction and activation status. Intensity of red color in molecules indicates expression levels as shown in the legend. B) Table with prediction confidence and activation z-score value for each molecule.
