# Supplementary material for: Proliferative arrest induces neuronal differentiation and innate immune responses in normal and Creutzfeldt-Jakob Disease agent (CJ) infected rat septal neurons
Source: PLoS One. 2025 May 28;20(5):e0323825. doi: 10.1371/journal.pone.0323825 (PMC12118874; doi:10.1371/journal.pone.0323825)
Supplement: S4 Fig — Shows enrichment of molecular signatures from two group comparison between Arst/Nl vs Prol/Nl groups. (DOCX) [file pone.0323825.s004.docx]

**S4 Fig.:** **Gene set enrichment analysis (GSEA) plots.** Shows enrichment of molecular signatures from two group comparison between Arst/Nl vs Prol/Nl groups.
