# Supplementary material for: Proliferative arrest induces neuronal differentiation and innate immune responses in normal and Creutzfeldt-Jakob Disease agent (CJ) infected rat septal neurons
Source: PLoS One. 2025 May 28;20(5):e0323825. doi: 10.1371/journal.pone.0323825 (PMC12118874; doi:10.1371/journal.pone.0323825)
Supplement: S5 Fig — Overrepresentation analysis was done on g:Profiler web server and enriched pathways (FDR p < 0.05) were clustered using Enrichment map application on Cytoscape. Gene list to the left shows common upregulated genes across those enriched pathways. (DOCX) [file pone.0323825.s005.docx]

**S5 Fig.: Example of Wnt signaling in arrested CJ+ cells.** Overrepresentation analysis was done on g:Profiler web server and enriched pathways (FDR p<0.05) were clustered using Enrichment map application on Cytoscape. Gene list to the left shows common upregulated genes across those enriched pathways.
