# Supplementary material for: Proliferative arrest induces neuronal differentiation and innate immune responses in normal and Creutzfeldt-Jakob Disease agent (CJ) infected rat septal neurons
Source: PLoS One. 2025 May 28;20(5):e0323825. doi: 10.1371/journal.pone.0323825 (PMC12118874; doi:10.1371/journal.pone.0323825)
Supplement: S6 Fig — Overlap between differentially expressed genes and anti-viral response induced by IFN. Activation is indicated by orange lines, and cellular location of the response genes are also shown. In CJ+ cells the IFNs are not extracellular in origin but made by the cell since no other cells are present to produce activating cDNAs. From RT/qPCR direct experimental results in CJ+ cells IFNs were far higher than in uninfected cells indicating above transcripts were enhanced and due to recrudescence of the infectious agent. (DOCX) [file pone.0323825.s006.docx]

**S6 Fig.:** **CJ+ cells: Ingenuity pathway analysis network**. Overlap between differentially expressed genes and anti-viral response induced by IFN. Activation is indicated by orange lines, and cellular location of the response genes are also shown. In CJ+ cells the IFNs are not extracellular in origin but made by the cell since no other cells are present to produce activating cDNAs. From RT/qPCR direct experimental results in CJ+ cells IFNs were far higher than in uninfected cells indicating above transcripts were enhanced and due to recrudescence of the infectious agent
