# Supplementary material for: Proliferative arrest induces neuronal differentiation and innate immune responses in normal and Creutzfeldt-Jakob Disease agent (CJ) infected rat septal neurons
Source: PLoS One. 2025 May 28;20(5):e0323825. doi: 10.1371/journal.pone.0323825 (PMC12118874; doi:10.1371/journal.pone.0323825)
Supplement: S7 Fig — Note the difference for the CJ + /CJ− comparison lane 3 with Arst/Nl in lane 1 and CJ + /Nl showing underlying differences in CJ− brought out by arrest. Blue color indicates a negative z-score (inhibition), while orange color indicates a positive z-score and activation. (DOCX) [file pone.0323825.s007.docx]

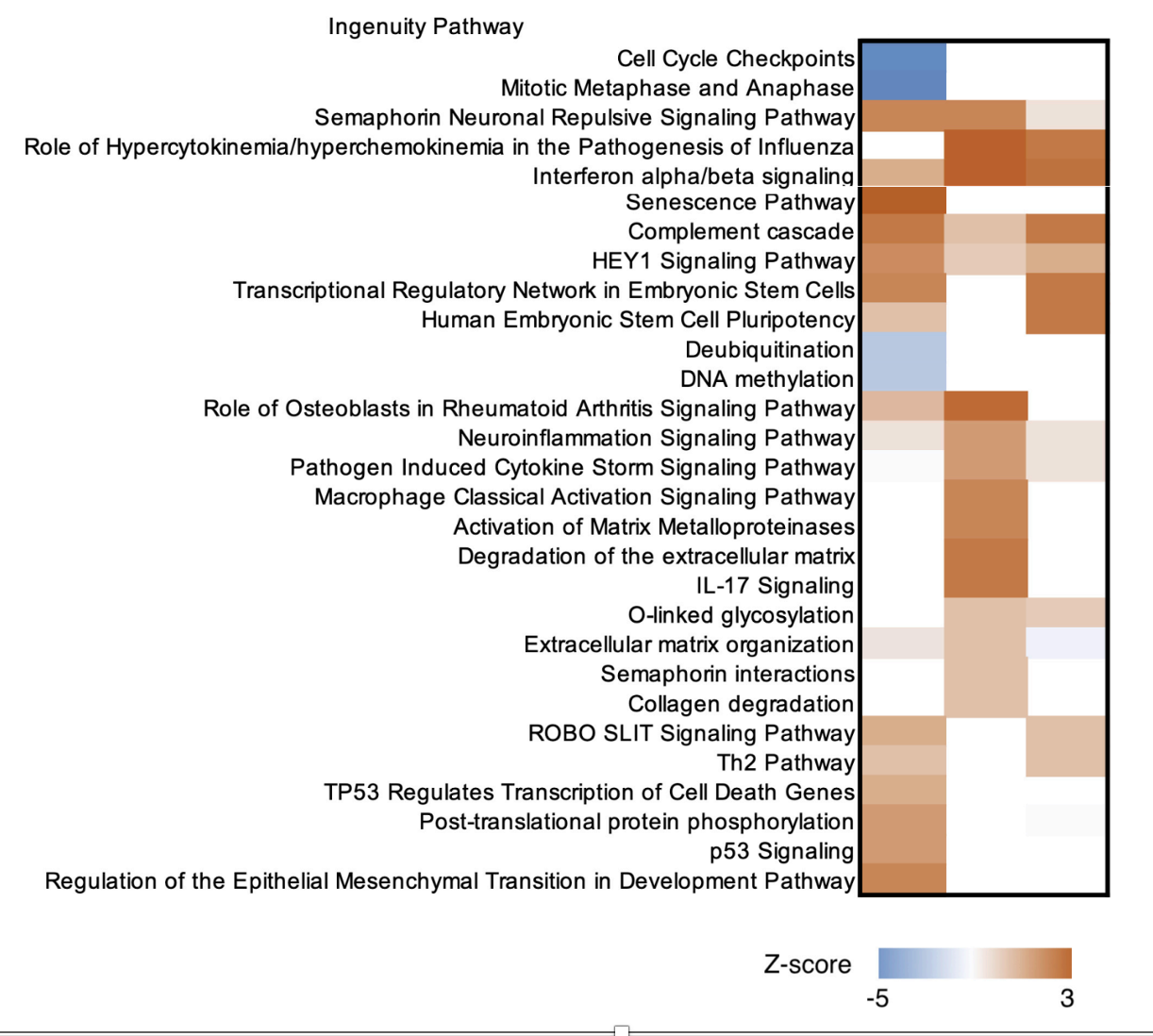


Ar-Nl/Nl CJ+/Nl CJ+/CJ-

**IPA path comparisons: Arst vs Prolif**

1) Arst/Nl vs Prol/Nl 2) Arst/CJ+ vs Prol/Nl 3) Arst/CJ+ vs Prol/ CJ-

**S7 Fig.: Ingenuity pathway analysis of three different comparisons based on pathway activation status (z-score).** Note the difference for the CJ+/CJ- comparison lane 3 with Arst/Nl in lane 1 and CJ+/Nl showing underlying differences in CJ- brought out by arrest. Blue color indicates a negative z-score (inhibition), while orange color indicates a positive z-score and activation.
