# Supplementary material for: Proliferative arrest induces neuronal differentiation and innate immune responses in normal and Creutzfeldt-Jakob Disease agent (CJ) infected rat septal neurons
Source: PLoS One. 2025 May 28;20(5):e0323825. doi: 10.1371/journal.pone.0323825 (PMC12118874; doi:10.1371/journal.pone.0323825)
Supplement: S8 Fig — Shows enriched molecular signatures comparing CJ+ versus Prol/Nl samples. Compare S4 above for gene enrichment analyses of Arst/Nl versus Prol/Nl. (DOCX) [file pone.0323825.s008.docx]

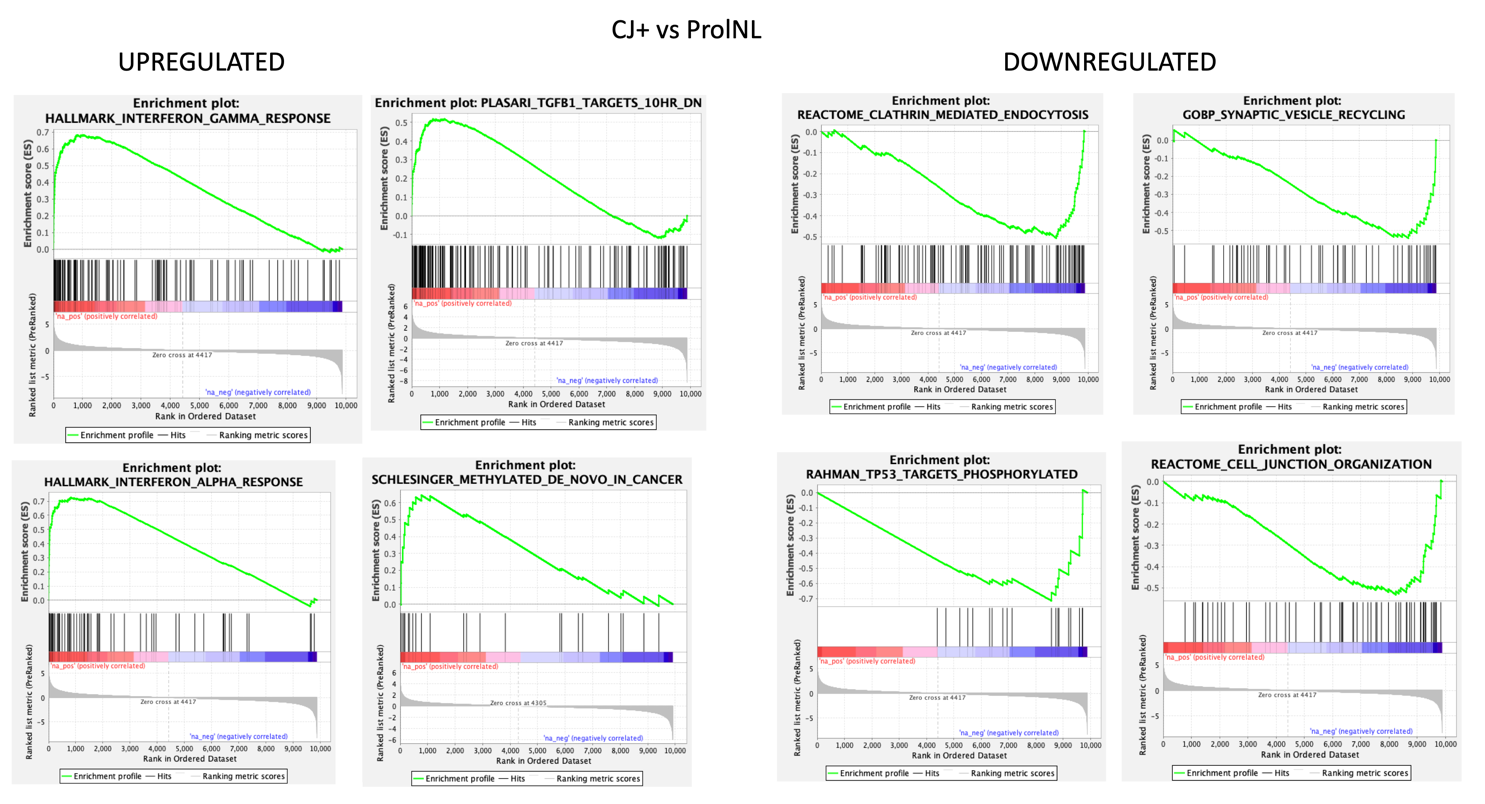


**S8 Fig.: Enrichment plots from Gene Set Enrichment Analysis (GSEA)**. Shows enriched molecular signatures comparing CJ+ versus Prol/Nl samples. Compare S4 above for gene enrichment analyses of Arst/Nl versus Prol/Nl.
