# Supplementary material for: Proliferative arrest induces neuronal differentiation and innate immune responses in normal and Creutzfeldt-Jakob Disease agent (CJ) infected rat septal neurons
Source: PLoS One. 2025 May 28;20(5):e0323825. doi: 10.1371/journal.pone.0323825 (PMC12118874; doi:10.1371/journal.pone.0323825)
Supplement: S9 Fig — B shows 146 CJ+ unique down regulated genes. Please refer to the supplement S10 for complete list. (DOCX) [file pone.0323825.s009.docx]

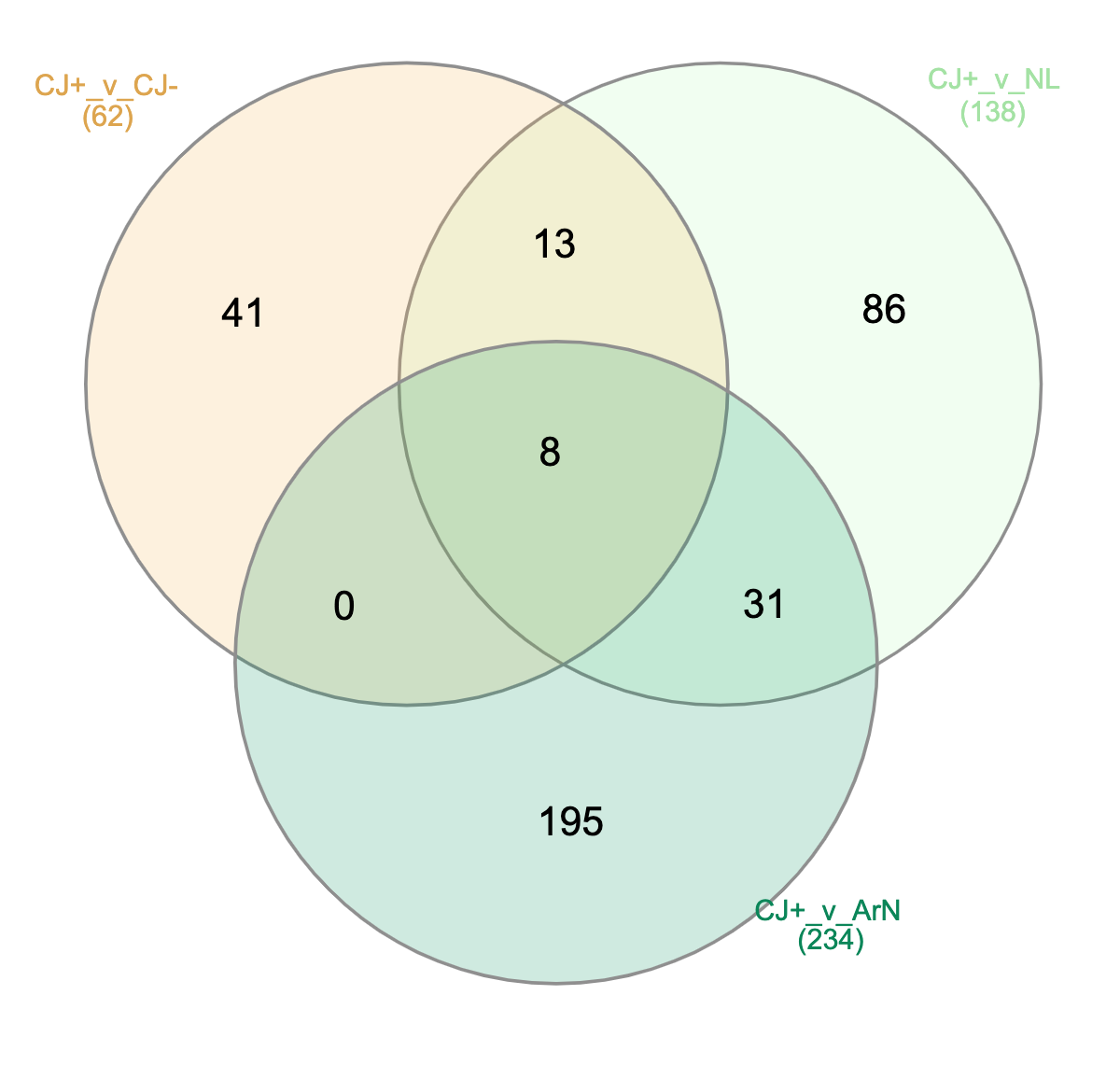

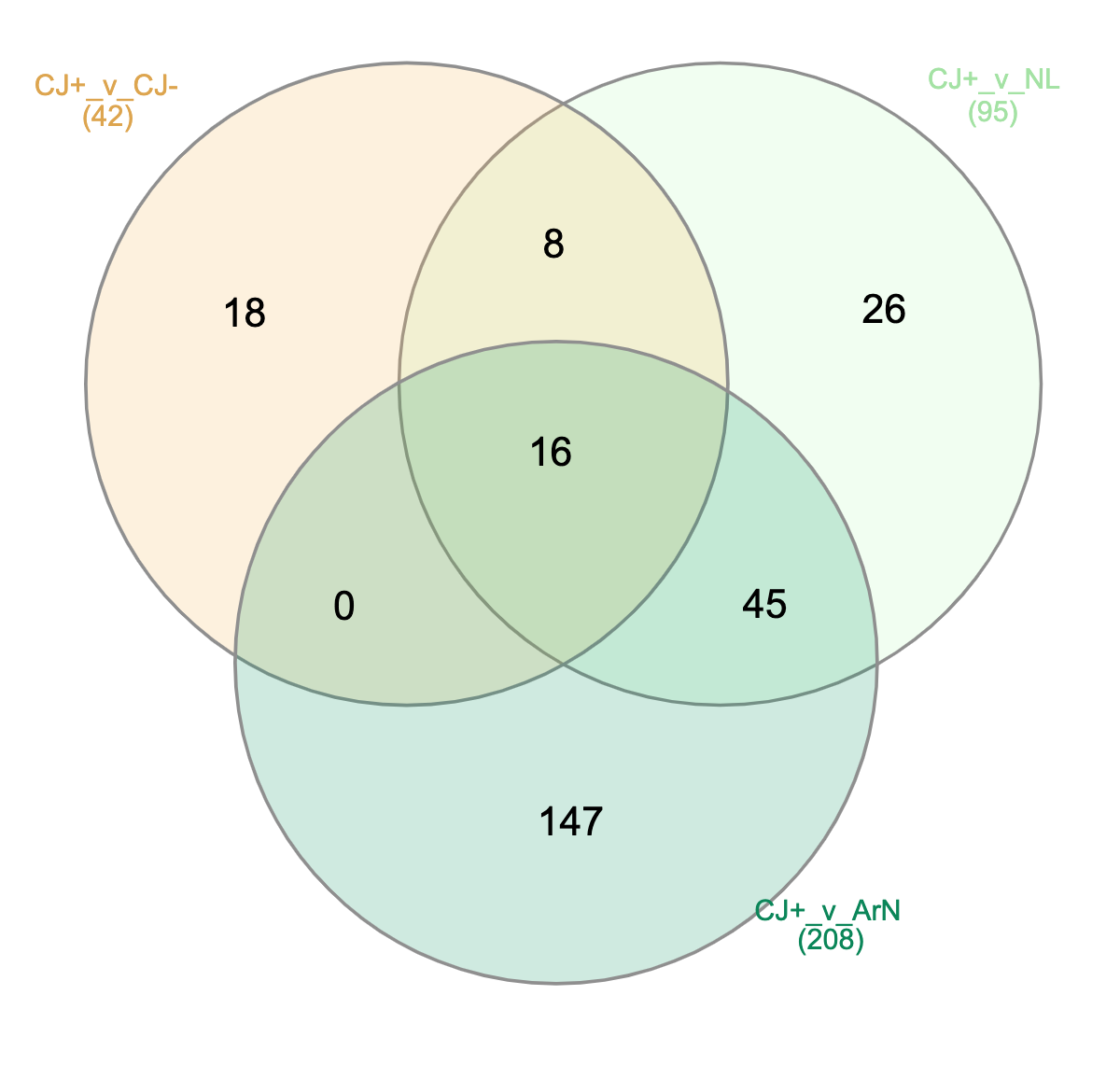


1. B

**S9 Fig.: Venn diagram of unique 196 upregulated CJ+ unique genes** (A) and 146 downregulated genes. B shows 146 CJ+ unique down regulated genes. Please refer to the supplement S10 for complete list.
