# Supplementary material for: Proliferative arrest induces neuronal differentiation and innate immune responses in normal and Creutzfeldt-Jakob Disease agent (CJ) infected rat septal neurons
Source: PLoS One. 2025 May 28;20(5):e0323825. doi: 10.1371/journal.pone.0323825 (PMC12118874; doi:10.1371/journal.pone.0323825)
Supplement: Attachment — Submitted filename: Manuscript Number PONE-D-24-53655.docx [file pone.0323825.s011.docx]

**General Comments**

This study investigates the impact of proliferative arrest on neuronal differentiation and innate immune responses in rat septal neurons, comparing normal cells to those infected with the Creutzfeldt-Jakob Disease (CJD) agent. It employs transcriptomic analysis to uncover changes associated with latent and productive CJD infection states. The findings highlight that proliferative arrest in normal neurons leads to upregulation of neuronal differentiation markers, anti-proliferative genes, and innate immune pathway genes.

In contrast, neurons with latent (CJ-) infection exhibit epigenetic imprinting without significant transcriptional differences from normal neurons. However, productive (CJ+) arrested neurons show suppressed neuronal differentiation, heightened interferon and cytokine responses, partial escape from proliferative control, and persistent high infectivity. The study proposes that latent CJD infections can cause lasting transcriptional and epigenetic changes, contributing to the silent persistence of infectious agents in peripheral tissues.

While the study is intriguing and relevant, it requires additional validation to strengthen its conclusions and enhance its suitability for publication.

**Major Comments**

1. **Lack of Functional Validation for Transcriptomic Findings**
   The manuscript provides valuable transcriptomic data but lacks functional or biochemical validation of key findings. While reproducibility across biological replicates is mentioned, RNA-seq results need to be validated using methods such as RT-qPCR or Western blot. RNA-level changes do not always correlate with protein-level changes or cellular phenotypes, making functional validation essential. To strengthen the robustness of the bioinformatics conclusions, the authors should validate the expression levels of selected genes with significant fold changes using RT-qPCR or similar methods.
2. **Restricted Focus on a Single CJD Strain and In Vitro Model**
   The study's focus on a single CJD strain (FU-CJD) limits the generalizability of its findings. Including additional CJD strains in the analysis would provide broader insights and enhance the study's applicability. Similarly, reliance solely on an in vitro cell culture model may not fully recapitulate in vivo conditions. Expanding the scope of the study to include other models or strains is recommended.

**Minor Comments**

1. **Abstract Revision**
   The abstract should be concise and easily understandable. The current abstract is overly complex and includes unnecessary details, such as specific candidate gene names, which are better suited for the main text. Simplifying the abstract will make it more accessible to readers.
2. **Discussion Length**
   The discussion section is excessively long (six pages), with repetitive elaboration on results. A discussion should provide a concise summary of the findings, their implications, and their alignment with existing literature. The authors should restructure this section to avoid redundancy and improve clarity.
