# Supplementary material for: Proliferative arrest induces neuronal differentiation and innate immune responses in normal and Creutzfeldt-Jakob Disease agent (CJ) infected rat septal neurons
Source: PLoS One. 2025 May 28;20(5):e0323825. doi: 10.1371/journal.pone.0323825 (PMC12118874; doi:10.1371/journal.pone.0323825)
Supplement: Attachment — Submitted filename: response_Manuscript Number PONE-D-24-53655.docx [file pone.0323825.s013.docx]

We have made changes in the manuscript that address and clarify every point made by reviewers as detailed below. In addition, we made substantial changes in the introduction, discussion and conclusions to simplify the flow and logic of the findings. While redundancies were cut, added explanatory changes further highlighted 1) relevant corroborating animal experiments showing early brain upregulation of cytokine/chemokines and TGF-α transcripts in response to CJ brain infection (**p21**, first paragraph), 2) IFN linked secondary transcripts in CJ+ microglia and 3) extensive prior biological reversible DNA replication, neurodifferentiation, and Western blot and RT/qPCR assays in our publications that predicted most of the secondary global changes found here. We thank the reviewers for their queries and comments that helped improve the manuscript. All required formatting changes, including supplemental and figure captions have also been made.

**Reviewer 1:**

**Major Comments**

1. **Lack of Functional Validation for Transcriptomic Findings**
   The manuscript provides valuable transcriptomic data but lacks functional or biochemical validation of key findings. While reproducibility across biological replicates is mentioned, RNA-seq results need to be validated using methods such as RT-qPCR or Western blot. RNA-level changes do not always correlate with protein-level changes or cellular phenotypes, making functional validation essential. To strengthen the robustness of the bioinformatics conclusions, the authors should validate the expression levels of selected genes with significant fold changes using RT-qPCR or similar methods.

RESPONSE: Major functional changes were assayed in these samples before doing transcriptional library analyses as published (4) with Western blot and RT/qPCR analyses (with detailed primers and methods) ***for over 70 different sequentially collected samples***. We now further clarify this point by referencing our previous study more explicitly in the re-written introduction and discussion and just before the materials and methods section. From the initial submission and still retained in the revised manuscript (lines from original submission):

*Line 114 “SEP cells… stop synthesizing DNA by 4-6 days post-arrest, as assessed by BrdU incorporation in nuclei, and these cells can be maintained in an arrested state by refeeding every 2 days for >75 days without reverting to a proliferative state (4). During this time, cell-to-cell contacts and neural markers increase along with host prion protein (PrP) assayed on western blots, and host gene transcripts (Prnp) assayed by RT/qPCR (4, 5).”*

*Line 122 “Previous RT/qPCR and Western blot protein studies of normal SEP cells were limited previously documented virtually complete cell arrest by cessation of nuclear DNA synthesis using labeled nucleotides, in addition to concomitant neurodifferentiation. For example, uninfected neurons exhibited a few chosen20 to 28-fold upregulation in Prnp expression by RT/qPCR with a sustained 8-fold increase in PrP on Western blots (4).*

*Line 128* “*These previous RT/qPCR studies of normal SEP neurons also showed that arrest induced an 8-fold upregulation of ß-interferon (ß-IFN) RNA, an unexpected classical innate immune response (4) not previously shown to be produced by normal neurons.”*

**Now additionally added (p5)** *“Prior detailed biological and molecular arrest studies including DNA synthesis, RT/qPCR and Western blots on these and other independent SEP cell experiments have been detailed previously (3, 4, 5).” Furthermore, actual amounts (fold) of previously validated RT/qPCR transcripts such as IFN-β e.g., “48 to 60-fold upregulated in CJ+ cells” now given (****p12****) along with others for OAS and PrnP RNAs.*

We also note that several unique new genes not obviously related to the previously validated replication, neurodifferentiation and IFN transcripts are of interest for further investigation (**p16**):*“Other unique transcripts identified* *here that may participate in CJ latency and pathology can now be tested in different cell and animal disease models.”*

2. **Restricted Focus on a Single CJD Strain and In Vitro Model**
   The study's focus on a single CJD strain (FU-CJD) limits the generalizability of its findings. Including additional CJD strains in the analysis would provide broader insights and enhance the study's applicability. Similarly, reliance solely on an in vitro cell culture model may not fully recapitulate in vivo conditions. Expanding the scope of the study to include other models or strains is recommended.

We previously noted this (massive) study was limited to a single strain (line 522). To put this in biological perspective **we now added:**

“*We examined only a single human TSE agent-strain; other agents such as sheep derived scrapie, BSE and cervid TSE agents might not show an identical pattern of responses. However, the fundamental biology of all TSE agents (modes of agent spread, latency, and basic brain pathology) are common to all TSE infections, and different agent- strains are likely to show only minor differences in the degree of change”*

Additionally (**p20**) *“in CJD infected mouse brains, the IFN linked transcripts IFI204, IFI 202, CXCL10 and IFR8 by RT/qPCR were elevated as early as 10-40 days post-inoculation, a time of progressive agent doubling (46).”*

Moreover, microglia also showed comparable related IFN-linked responses as stated on p20 last paragraph: *“CJ brain infection activates myeloid microglia and…..isolated microglia displayed several IFN regulated and activated genes, e.g., IFI204, IRF2 and IRF8 and… additional immune and inflammatory responses were brought out by poly I:C (e.g., ISG15, CXCL10 and OAS) stimulation (45)… correspond to the CJ+ SEP transcripts identified here, suggesting a common response to infection by very different cell types.*”

**Minor Comments**

1. **Abstract Revision**
   The abstract should be concise and easily understandable. The current abstract is overly complex and includes unnecessary details, such as specific candidate gene names, which are better suited for the main text. Simplifying the abstract will make it more accessible to readers.

We clarified the abstract and have removed some of the less central gene names.

2. **Discussion Length**
   The discussion section is excessively long (six pages), with repetitive elaboration on results. A discussion should provide a concise summary of the findings, their implications, and their alignment with existing literature. The authors should restructure this section to avoid redundancy and improve clarity.

We have restructured the discussion section and removed redundant elaborations to provide a more concise and focused analysis of important findings and additional inserts above. Now it is much easier to understand.

**Reviever 2:**

This manuscript by Pagano et al. investigated how RNA expression varies in rat post-mitotic septal neuronal cell lines at arrested versus proliferative states in both infected and uninfected conditions. The study identifies several pathways that are associated with specific states and conditions which can provide the basis for future studies into the mechanisms of neural differentiation as well immune responses in neurodegenerative diseases. The study was rigorously performed and the findings are well presented. I suggest some minor modifications:
1. The authors need to always provide the full form of terms while introducing them for the first time.

We added the full form of terms when first introduced.

2. The authors need to provide a protocol for qPCRs in the materials and methods section and provide a list of all the primers used along with their sequences.

As noted above, we reference our previous study (containing the protocol for qPCR and a list of all primers) just before the beginning of the materials and methods section.

3. The figures have been written as figure.tif at multiple occasions. The authors need to correct that. Also, the authors often tended to start sentences by 'The figure shows' which is a rather uncommon practice. Its better to just state the findings and mention the relevant figure in bracket.

The figure titles were corrected.

4. The introduction requires some rewriting to improve clarity about the necessity of this endeavor. It might be better if the authors start with the disease aspect of this study and make a logical progression to justify the usage of SEP cells for this study. Then they can provide information about relevant studies in these cells and then proceed to the next section.

We modified the introduction to clarify the relevance of latency of infection and brain disease, that are clearly central for human CJD infections.
